# The GW/BSE Method in Magnetic Fields

**DOI:** 10.3389/fchem.2021.746162

**Published:** 2021-11-25

**Authors:** Christof Holzer, Ansgar Pausch, Wim Klopper

**Affiliations:** ^1^ Institute of Theoretical Solid State Physics, Karlsruhe Institute of Technology (KIT), Karlsruhe, Germany; ^2^ Institute of Physical Chemistry, Karlsruhe Institute of Technology (KIT), Karlsruhe, Germany; ^3^ Institute of Nanotechnology, Karlsruhe Institute of Technology (KIT), Eggenstein-Leopoldshafen, Germany

**Keywords:** GW, Bethe-Salpeter, excitation energy, magnetic field, density functional theory

## Abstract

The *GW* approximation and the Bethe–Salpeter equation have been implemented into the Turbomole program package for computations of molecular systems in a strong, finite magnetic field. Complex-valued London orbitals are used as basis functions to ensure gauge-invariant computational results. The implementation has been benchmarked against triplet excitation energies of 36 small to medium-sized molecules against reference values obtained at the approximate coupled-cluster level (CC2 approximation). Finally, a spectacular change of colour from orange to green of the tetracene molecule is induced by applying magnetic fields between 0 and 9,000 T perpendicular to the molecular plane.

## 1 Introduction

The description of excited states of molecules in strong magnetic fields poses a major challenge for quantum chemical methods. (Delos et al., 1983; Turbiner and López Vieyra, 2004; Hampe and Stopkowicz, 2017; Stopkowicz, 2018; Hampe and Stopkowicz, 2019; Wibowo et al., 2021). On the one hand, it is well known that introducing magnetic fields also introduces a gauge-dependence when standard, real-valued Gaussian-type basis functions are used. As a solution, as proposed by London, a complex phase factor countering the gauge-dependence of the magnetic field, can be used. (London, 1937; Helgaker and Jørgensen, 1991; Ruud et al., 1993; Tellgren et al., 2008). This in turn leads to complex-valued basis functions, which significantly increase the cost of subsequent calculations. On the other hand, many “work-horse” methods used to describe excited states as linear-response (LR) time-dependent density functional theory (TD-DFT) cannot be straightforwardly adapted to include arbitrary magnetic fields due to instabilities occurring in the respective non-collinear exchange-correlation (XC) kernel. The instabilities in the XC kernel are related to the same instabilities that also plague other non-collinear TD-DFT kernels in, for example, relativistic two-component TD-DFT. (Gao et al., 2005; Egidi et al., 2017; Komorovsky et al., 2019). While solutions to these problems have been proposed, they inevitably lead to XC kernels that do not exhibit full rotational invariance. (Egidi et al., 2017; Komorovsky et al., 2019). Contrary to TD-DFT, coupled-cluster methods are not plagued by any instabilities, but suffer from their steep cost, which increases exponentially with their accuracy. Furthermore, the complex gauge-independent London atomic orbitals lead to another steep increase in the computational complexity, effectively preventing calculations on systems with more than a few electrons. (Hampe and Stopkowicz, 2017; Hampe et al., 2020). Even though the computational limitations are severe, the investigation of molecular properties in strong external magnetic fields has become an increasingly popular topic within the field of quantum chemistry in recent years. Several field-dependent properties including non-linear effects on the electronic structure of small molecules, (Tellgren et al., 2008; Tellgren et al., 2009; Lange et al., 2012; Stopkowicz et al., 2015), molecular geometries, (Tellgren et al., 2012; Irons et al., 2021), spin-phase transitions (Sun et al., 2019a) and excited state properties (Sun et al., 2019b; Sen et al., 2019; Stetina et al., 2019; Wibowo et al., 2021) have been explored using quantum-chemical methods at different levels of theory.

Since the largest magnetic field currently created on Earth exhibits a field strength of about 100 T, (Sims et al., 2008), there is hardly any need to treat strong magnetic fields in more than a perturbative manner from an experimental point of view. Still, scientific curiosity has for a long time been a strong motor to investigate also situations which are (currently) not directly accessible. Given the lack of experimental data, highly accurate quantum-chemical methods are desirable in order to explore molecular properties in the field regime of >100 tesla.

With the *GW*/Bethe–Salpeter equation (BSE) method, a suitable way of calculating properties from Kohn-Sham (KS) reference states has emerged within the last few years. (Bruneval et al., 2015; Jacquemin et al., 2015; Leng et al., 2016; Holzer and Klopper, 2017; Krause and Klopper, 2017; Gui et al., 2018; Blase et al., 2020; Kehry et al., 2020). It has seen great success, exhibiting a more favourable behaviour than TD-DFT on many occasions. While both TD-DFT and the *GW*/BSE method start from the same Kohn-Sham reference, *GW*/BSE fully accounts for charge-transfer and Rydberg excitations due to its correct asymptotic long-range behaviour. (Sagmeister and Ambrosch-Draxl, 2009; Blase and Attaccalite, 2011; Blase et al., 2011; Blase et al., 2018). Furthermore, the description of core excitations is significantly improved within the *GW*/BSE method. (Olovsson et al., 2009; Vinson et al., 2011; Kehry et al., 2020). The accuracy of the *GW*/BSE method is an improvement over TD-DFT. Therefore, adapting the *GW*/BSE method to be applicable to arbitrary molecules in arbitrary magnetic fields is worthwhile. It allows for an investigation of the effects of strong magnetic fields in sizable molecular systems while still retaining a certain robustness with respect to accuracy.

Within this paper we therefore aim at describing a fully consistent formulation and implementation of the *GW*/BSE method for the description of optical spectra of sizable molecules within strong magnetic fields. In the following chapters, the general formulas for the *G*_0_ *W*_0_ and the eigenvalue self-consistent *GW* (ev*GW*) methods as well as the BSE in strong magnetic fields are outlined. The resulting implementation is able to describe excited states of molecules of significant size. As such strong external magnetic fields are not accessible in experimental setups, a set of benchmark values obtained from truncated coupled cluster theory is provided for 36 small to medium-sized molecules. Finally, we demonstrate the capabilities of the *GW*/BSE equation in strong magnetic fields by predicting the colour change of tetracene in a strong uniform magnetic field.

## 2 Theory

### 2.1 GW Approach in Magnetic Fields Using London Atomic Orbitals

*GW* quasiparticle (QP) energies form the basis for calculating excitation energies from the Bethe–Salpeter equation. The principal theory to obtain *GW* QP energies in a magnetic field has been outlined in Ref. (Holzer et al., 2019). for atoms and complex-valued spinors. For molecules, to retain full gauge-invariance, instead of real Gaussian-type atomic orbitals, complex London-type atomic orbitals (LAOs) have to be used. These are obtained as a direct product of a Gaussian-type orbital *ϕ*_*μ*_(**r**) and a complex phase factor:

$$\xi_{\mu }\left(r\right)=\phi_{\mu }\left(r\right)e^{-i\;k_{\mu }\cdot r}$$ (1a)

$$k_{\mu }=\frac{1}{2}B\times \left(R_{\mu }-O\right)$$ (1b)

The complex phase factor is used in order to cancel the dependency of all observable properties on the gauge origin **O** which naturally arises from the choice of a Coulomb gauge (∇ ⋅**A** = 0) for a magnetic vector potential (∇ ×**A** = **B**). In a two-component (2c) framework, complex spinors can be constructed as a linear combination of LAOs:

$$\left|p\right\rangle \rangle =\varphi_{p}\left(x\right)=\underset{\mu }{\sum }\left\{C_{\mu p}^{\alpha }\xi_{\mu }\left(r\right)\alpha \left(\sigma \right)+C_{\mu p}^{\beta }\xi_{\mu }\left(r\right)\beta \left(\sigma \right)\right\}.$$ (2)

Non-collinear spin densities are well represented in this 2c spinor framework. Therefore, uniform and non-uniform magnetic fields can be included in this way. (Sen et al., 2019). More generally, within the notation used in this paper, any arbitrary non-collinear spin density can be employed. Furthermore, the complex phase-factor including LAOs are strictly needed to ensure gauge-independence for *GW* quasiparticle energy evaluations of multi-atomic systems, as well as for consecutive calculations of excitation energies using the Bethe–Salpeter equation. As the magnetic field is represented by a one-electron operator within 2c Kohn–Sham equations, the according information is fully absorbed into the complex spinors expanded in LAOs. Therefore, all quantities occurring in the BSE in a magnetic field must generally be assumed to be complex, unless further symmetries can be exploited.

To obtain the working formulas for *G*_0_ *W*_0_ and ev*GW*, we closely follow Refs. (Holzer et al., 2019). and (Hedin, 1991) and define the charge-fluctuation potential as

$$V_{m}\left(x\right)=\int_{-\infty }^{\infty }\frac{1}{\left|r-r^{\prime }\right|}\rho_{m}\left(x^{\prime }\right)\;dx^{\prime },$$ (3)

where *m* denotes an excited state, and where the space-spin-coordinate **x** ≡ (**r**, *σ*) includes both space and spin coordinates. The charge fluctuation can be expressed using molecular spinors as

$$\rho_{m}\left(x\right)=\underset{ia}{\sum }\left[\varphi_{a}^{*}\left(x\right)\varphi_{i}\left(x\right)X_{ia}^{m}+\varphi_{i}^{*}\left(x\right)\varphi_{a}\left(x\right)Y_{ia}^{m}\right],$$ (4)

where $X_{ia}^{m}$ ( $Y_{ia}^{m}$ ) refers to the elements “*ia*” of the *m*th column of the matrix **X** (**Y**) obtained from solving the direct random-phase approximation equation (dRPA) as defined by Equations 4–7 of Ref. (Holzer et al., 2019). Here and in the following, we use the indices *i*, *j*, *k*, … for occupied molecular spinors, *a*, *b*, *c*, … for unoccupied (virtual) molecular spinors, and *p*, *q*, *r*, … for arbitrary molecular spinors, expanded in a basis set of LAOs. It is worthwhile to note that complex molecular spinors can be obtained from London atomic orbitals as well as from real Gaussian orbitals, and after the transformation from an atomic to a molecular picture, the working equations are the same for the two basis sets. However, only molecular spinors from LAOs incorporate the information needed for proper gauge-invariant calculations.

In the *GW* approximation, the correlation self-energy is obtained from the expression

$$\begin{array}{cc} \Sigma_{c}\left(x,x^{\prime };\omega \right) & =-\frac{1}{2\pi i}\int_{-\infty }^{\infty }e^{i\omega^{\prime }0^{+}}W_{c}\left(x,x^{\prime };\omega^{\prime }\right)\\ & \times G\left(x,x^{\prime };\omega +\omega^{\prime }\right)d\omega^{\prime }, \end{array}$$ (5)

where *G* is the one-electron Green’s function

$$G\left(x,x^{\prime };\omega \right)=\underset{p}{\sum }\frac{\varphi_{p}\left(x\right)\varphi_{p}^{*}\left(x^{\prime }\right)}{\omega -\varepsilon_{p}+i\delta \mathrm{sgn}\left(\varepsilon_{p}-\mu \right)}.$$ (6)

As usual, to avoid instabilities and to make Eq. 6 integratable, a small positive number *δ* is added to the denominator. *ɛ*_*p*_ is the eigenvalue of the *p*th spinor that solves the Kohn-Sham equation for the underlying density functional approximation. The Fermi-level chemical potential *μ* is chosen to lie between the energy levels of the lowest unoccupied and highest occupied spinors, and *W*_*c*_ is the correlation contribution to the linearly screened potential,

$$W_{c}\left(x,x^{\prime };\omega \right)=\underset{m\neq 0}{\sum }\left[\frac{V_{m}\left(x\right)V_{m}^{*}\left(x^{\prime }\right)}{\omega -\omega_{m}+i\delta }-\frac{V_{m}^{*}\left(x\right)V_{m}\left(x^{\prime }\right)}{\omega +\omega_{m}-i\delta }\right].$$ (7)

Evaluating the integral on the right-hand side of Eq. 5 yields

$$\begin{array}{cc} \Sigma_{c}\left(x,x^{\prime };\omega \right) & =\underset{k}{\sum }\underset{m\neq 0}{\sum }\frac{V_{m}\left(x\right)V_{m}^{*}\left(x^{\prime }\right)\varphi_{k}\left(x\right)\varphi_{k}^{*}\left(x^{\prime }\right)}{\omega +\omega_{m}-\varepsilon_{k}-i\eta }\\ & +\underset{c}{\sum }\underset{m\neq 0}{\sum }\frac{V_{m}^{*}\left(x\right)V_{m}\left(x^{\prime }\right)\varphi_{c}\left(x\right)\varphi_{c}^{*}\left(x^{\prime }\right)}{\omega -\omega_{m}-\varepsilon_{c}+i\eta }, \end{array}$$ (8)

where *η* = 2*δ*. We thus obtain the following working equation for the real-valued correlation contribution to the quasiparticle energy:

$$\left\langle p|\Sigma_{c}\left(\varepsilon_{p}\right)|p\right\rangle =\underset{k}{\sum }\underset{m\neq 0}{\sum }|\left(pk|\rho_{m}\right){|}^{2}D_{p,k,m}^{+}+\underset{c}{\sum }\underset{m\neq 0}{\sum }|\left(cp|\rho_{m}\right){|}^{2}D_{p,c,m}^{-},$$ (9)

with

$$D_{p,q,m}^{\pm }\;=\;\frac{\varepsilon_{p}-\varepsilon_{q}\pm \omega_{m}}{{\left(\varepsilon_{p}-\varepsilon_{q}\pm \omega_{m}\right)}^{2}+\eta^{2}}.$$ (10)

The two-electron integrals (*pq*|*ρ*_*m*_) are computed as

$$\left(pq|\rho_{m}\right)=\underset{ia}{\sum }\left[\left(pq|ai\right)X_{ia}^{m}+\left(pq|ia\right)Y_{ia}^{m}\right].$$ (11)

The exchange self-energy is

$$\left\langle p|\Sigma_{x}|p\right\rangle =-\underset{k}{\sum }\left(pk|kp\right),$$ (12)

and the *G*_0_ *W*_0_ quasiparticle energies are computed as (van Setten et al., 2012; Krause et al., 2015; Holzer et al., 2019)

$$\varepsilon_{p}^{G_{0}W_{0}}=\varepsilon_{p}^{\left(0\right)}+Z_{p}\left\langle p|\Sigma_{c}\left(\varepsilon_{p}^{\left(0\right)}\right)+\Sigma_{x}-V_{\text{xc}}|p\right\rangle ,$$ (13)

with

$$Z_{p}={\left\{1-\left\langle p|{\left(\partial \Sigma_{c}\left(\varepsilon \right)/\partial \varepsilon \right)}_{\varepsilon =\varepsilon_{p}^{\left(0\right)}}|p\right\rangle \right\}}^{-1},$$ (14)

where *Σ*_X_ is the exchange self-energy and *V*_xc_ is the exchange-correlation potential of the underlying density functional theory. To obtain eigenvalue self-consistent quasiparticle energies (i.e., ev*GW* quasiparticle energies), Eq. 13 is evaluated repeatedly with *Z* = 1 until the obtained eigenvalues are converged.

$$\varepsilon_{p}^{\left(n+1\right)}=\varepsilon_{p}^{\left(0\right)}+\left\langle p|\Sigma_{c}\left(\varepsilon_{p}^{\left(n\right)}\right)+\Sigma_{x}-V_{\text{xc}}|p\right\rangle .$$ (15)

It was found that DIIS (direct inversion in the iterative subspace) (Pulay, 1980) procedures can speed up this process considerably. Usually less than ten consecutive evaluations of Eq. 15 are then needed to obtain converged ev*GW* quasiparticle energies.

Finally, we note that also the analytic continuation (AC) and contour deformation (CD) *GW* variants described in Ref. (Holzer and Klopper, 2019) can be adapted to LAOs in the same manner. However, unlike the previous formulas derived for the analytic *GW* variant in an magnetic field, our current AC-*GW* and CD-*GW* variants are approximate in the sense that they ignore the lack of time-reversal (Kramers) symmetry. While we expect our AC-*GW* and CD-*GW* variants to be well behaved in a system with a vanishing spin expectation value (⟨*S* ^2^⟩ ≈ 0), more research on these methods has to be performed in cases of non-vanishing ⟨*S* ^2^⟩.

### 2.2 The Bethe–Salpeter Equation in a Magnetic Field

Starting from the gauge-invariant quasiparticle energies described in the previous section, the gauge-invariant excitation energies can be obtained from the Bethe–Salpeter equation also making use of LAOs. The BSE can be expressed in terms of complex spinors as

$$\left(\begin{array}{cc} A & B\\ B^{*} & A^{*} \end{array}\right)\left(\begin{array}{c} X^{m}\\ Y^{m} \end{array}\right)=\omega_{m}\left(\begin{array}{cc} 1 & 0\\ 0 & -1 \end{array}\right)\left(\begin{array}{c} X^{m}\\ Y^{m} \end{array}\right).$$ (16)

The orbital rotation matrices **A** and **B** are defined as

$$A_{ia,jb}=\left(\varepsilon_{i}-\varepsilon_{a}\right)\delta_{ij}\delta_{ab}+v_{ai,bj}-W_{ji,ba},$$ (17a)

$$B_{ia,jb}=v_{ai,jb}-W_{bi,ja},$$ (17b)

where *ɛ*_*i*_ is the quasiparticle energy of the *i*th Kohn–Sham eigenstate from a preceding *GW* computation, *v*_*ia*,*bj*_ is a Coulomb integral over complex spinors,

$$v_{ia,bj}=\left(\varphi_{a}\varphi_{i}|\varphi_{b}\varphi_{j}\right)=\int \;\;\int \varphi_{a}^{*}\left(x\right)\varphi_{i}\left(x\right)\frac{1}{\left|r-r^{\prime }\right|}\varphi_{b}^{*}\left(x^{\prime }\right)\varphi_{j}\left(x^{\prime }\right)\;dxdx^{\prime },$$ (18)

and *W*_*pq*,*rs*_ is the static screened potential from the BSE. Properties such as for example oscillator strengths, excited state dipole moments, or nuclear forces can be obtained in a straightforward manner from the solutions of the eigenvalue problem of the BSE, again expressed in a basis of LAOs. Using complex LAO-based spinors, the static (*i.e.*, *ω* = 0) screened potential *W*, which is given in its real-space expression in Eq. 7, takes the form

$$W_{pq,rs}=\underset{tu}{\sum }{\left(\epsilon^{-1}\right)}_{pq,tu}v_{tu,rs}$$ (19a)

$$\epsilon_{pq,tu}=\delta_{pt}\delta_{qu}-\underset{tu}{\sum }v_{pq,tu}{\left(\chi_{0}\right)}_{tu,tu}\;,$$ (19b)

where ϵ is the dielectric function. The non-interacting response-function *χ*_0_ is diagonal and real if the quasiparticle energies are real, even if complex LAO-based spinors are used:

$${\left(\chi_{0}\right)}_{tu,tu}=\underset{kc}{\sum }\frac{\delta_{tk}\delta_{uc}+\delta_{tc}\delta_{uk}}{\varepsilon_{k}-\varepsilon_{c}}\;.$$ (20)

From the response function, and using the resolution-of-the-identity (RI) approximation

$$v_{pq,rs}=\underset{P}{\sum }{\left(R_{pq}^{P}\right)}^{*}R_{rs}^{P},$$ (21)

the screened potential *W*_*pq*,*rs*_ can be evaluated as (Krause and Klopper, 2017)

$$W_{pq,rs}=\underset{PQ}{\sum }{\left(R_{pq}^{P}\right)}^{*}{\left[\delta_{PQ}-2R\underset{ck}{\sum }R_{ck}^{P}{\left(\chi_{0}\right)}_{ck,ck}{\left(R_{ck}^{Q}\right)}^{*}\right]}^{-1}\;\;\;\;R_{rs}^{Q}\;.$$ (22)

For the 3-index intermediate $R_{pq}^{P}$

$$R_{pq}^{P}=\underset{Q}{\sum }{\left(V^{-1/2}\right)}_{PQ}\left(\phi_{Q}|\varphi_{p}\varphi_{q}\right),$$ (23a)

$$V_{PQ}=\left(\phi_{P}|\phi_{Q}\right)=\int \;\;\int \phi_{P}\left(r\right)\frac{1}{\left|r-r^{\prime }\right|}\phi_{Q}\left(r^{\prime }\right)\;drdr^{\prime }\;,$$ (23b)

$$\left(\phi_{Q}|\varphi_{p}\varphi_{q}\right)=\int \;\;\int \phi_{Q}\left(r\right)\frac{1}{\left|r-r^{\prime }\right|}\varphi_{p}^{*}\left(x^{\prime }\right)\varphi_{q}\left(x^{\prime }\right)\;drdx^{\prime },$$ (23c)

the auxiliary functions *ϕ*_*P*_ are chosen to be real as ordinary Gaussian-type atomic orbitals without losing gauge invariance of the results obtained from computations in a magnetic field. This considerably simplifies the inner part of Eq. 22, representing the response function in the auxiliary subspace,

$$\chi_{PQ}=\delta_{PQ}-2R\underset{ck}{\sum }R_{ck}^{P}{\left(\chi_{0}\right)}_{ck,ck}{\left(R_{ck}^{Q}\right)}^{*},$$ (24)

which is symmetric and real for the special case of the static BSE even in a (uniform or nonuniform) magnetic field. Finally, the efficient evaluation of the 3-index integrals (*ϕ*_*Q*_|*φ*_*p*_ *φ*_*q*_) has been described in Ref. (Pausch and Klopper, 2020). Therefore, the evaluation of the BSE in magnetic fields can proceed in a straightforward manner, making it an invaluable tool to assess excited stats of molecules in magnetic fields at roughly the same cost as required for linear-response Hartree–Fock computations, with the advantage of being significantly more accurate.

## 3 Computational Details

All implementation work in this work have been carried out in the framework of the Turbomole (Ahlrichs et al., 1989; Furche et al., 2014; Balasubramani et al., 2020) program package. Consequently, all calculations have also been done using Turbomole.

To assess the correctness of the implementation and theory of the *GW*/BSE method in magnetic fields, the first excited triplet states of 36 small molecules have been evaluated using ev*GW*/BSE in a magnetic field of 1,000 T in *z*-direction with respect to the coordinates supplied in the Supporting Information (SI). The molecules used in this evaluation are acetaldehyde, acetylene, CCl_2_, CClF, CF_2_, cyanoacetylene, cyanoformaldehyde, cyanogen, diacetylene, difluorodiazirine, formaldehyde, formic acid, formyl chloride, formyl fluoride, glyoxal, H_2_C_3_, HCN, HCP, HNO, HPO, HPO, HPS, HSiF, isocyanogen, nitrosamine, nitrosylcyanide, phosgene, propynal, pyrazine, selenoformaldehyde, SiCl_2_, silylidene, tetrazine, thioformaldehye, thioformylchloride, thionylcarbonylfluoride, and thiophosgene. The set of molecules is taken from Ref. (Suellen et al., 2019). Starting from the geometries provided in Ref. (Suellen et al., 2019), the geometries have been re-optimized using RI-MP2 (resolution-of-identity Møller–Plesset perturbation theory to second order) in the corresponding magnetic field of 1,000 T in *z*-direction using numerical gradients. At the RI-MP2-optimized geometries, the three first excited states have been evaluated using ev*GW*/BSE and PBE0, (Perdew et al., 1997; Adamo and Barone, 1999), LC-*ω*PBE, (Vydrov and Scuseria, 2006), BHLYP (also known as BH&HLYP), (Becke, 1993), and CAM-B3LYP (Yanai et al., 2004) as underlying functionals. In all calculations the energy and norm of the difference density matrix were converged to 10^−8^ hartree and 10^−7^, respectively. The def2-TZVP basis set (Weigend and Ahlrichs, 2005) was used throughout, in conjunction with the resolution-of-identity (RI) approximation for the Hartree and exchange terms with the corresponding auxiliary fitting basis sets for the Kohn–Sham ground-state (Weigend, 2006; Weigend, 2008) as well as the appropriate auxiliary fitting basis sets for the RI-MP2 and GW/BSE calculations. (Hättig, 2005).

For further comparison, the corresponding excited state energies have also been determined using the approximate coupled-cluster RI-CC2 method, (Hättig and Weigend, 2000), which has been adapted to calculations in finite magnetic fields in the course of the present work. It is closely related to the equation-of-motion coupled-cluster singles-and-doubles (EOM-CCSD) method in magnetic fields that has been described by Hampe and Stopkowicz, (Hampe and Stopkowicz, 2017), and to the two-component RI-CC2 implementation of Krause and Klopper. (Krause and Klopper, 2015). Compared to EOM-CCSD, RI-CC2 is computationally significantly less involved. This allows for the assessment of the larger molecules in the test set in the applied magnetic field.

For tetracene, we performed calculations on the ev*GW* (10)/BSE@DFT level using the contour deformation (CD) technique. (Holzer and Klopper, 2019). CD-ev*GW* (10) denotes that only the highest 10 occupied and lowest 10 unoccupied spinor energies have been corrected using CD-ev*GW*, while the remaining spinor energies are shifted (“scissoring”) accordingly. Testing the self-energy obtained from the CD-*GW* variant reveals that indeed for the systems and magnetic field strengths investigated in this paper, CD-*GW* exhibits errors of the order of 1 meV or less, making it perfectly feasible for (not too strong) magnetic fields. As reference density functionals for the ev*GW*(10)/BSE@DFT calculations, we used PBE0, B3LYP, BHLYP and CAM-B3LYP. Also the tetracene calculations were carried out in the def2-TZVP basis.

## 4 Results and Discussion

### 4.1 Test Set of Small Molecules

For the 36 molecules tested, in a field of 1,000 T, all ground states retain their closed-shell character, yielding no spin polarization. Therefore, the spacing in-between the three triplet states (T_−1,0,1_), which are non-degenerate in the magnetic field, are solely determined by the Zeeman effect. The T_−1,1_ components of the triplet are found exactly at $E_{T_{0}}\pm B$ . At a field of *B* = 1,000  tesla, this translates into ≈ ± 0.116 eV above and below the T_0_ state. The center-of-mass of the triplet, being located exactly at the zero-component of the triplet, is however shifted when compared to the degenerate triplet state in the field-free case.

**FIGURE 1:**
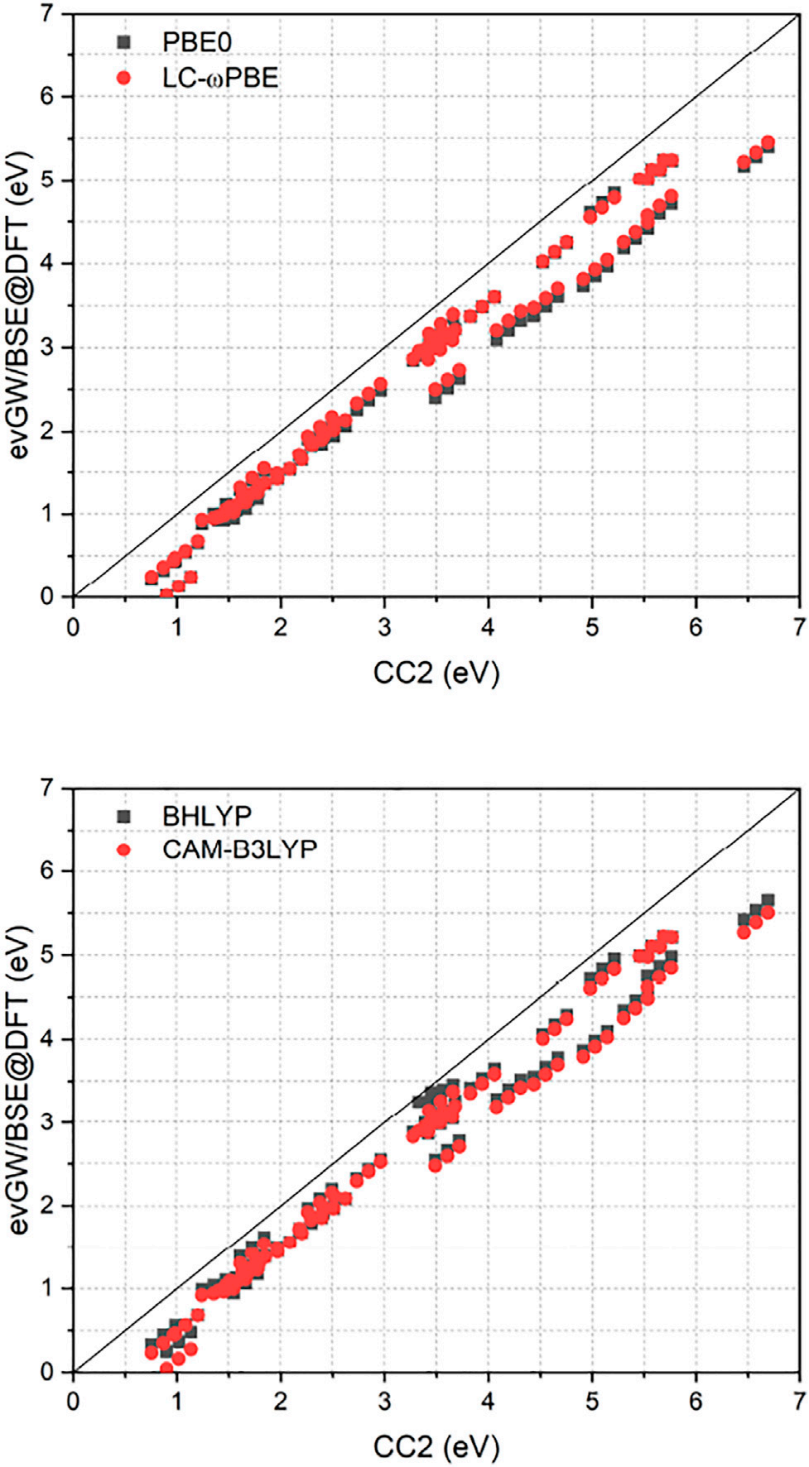
Comparison of excitation energies from the RI-CC2 and ev*GW*/BSE@DFT methods. ev*GW*/BSE calculations have been performed using either PBE0, LC-*ω*PBE, BHLYP or CAM-B3LYP spinors. All calculations used the def2-TZVP basis set. All values in eV.

**FIGURE 2:**
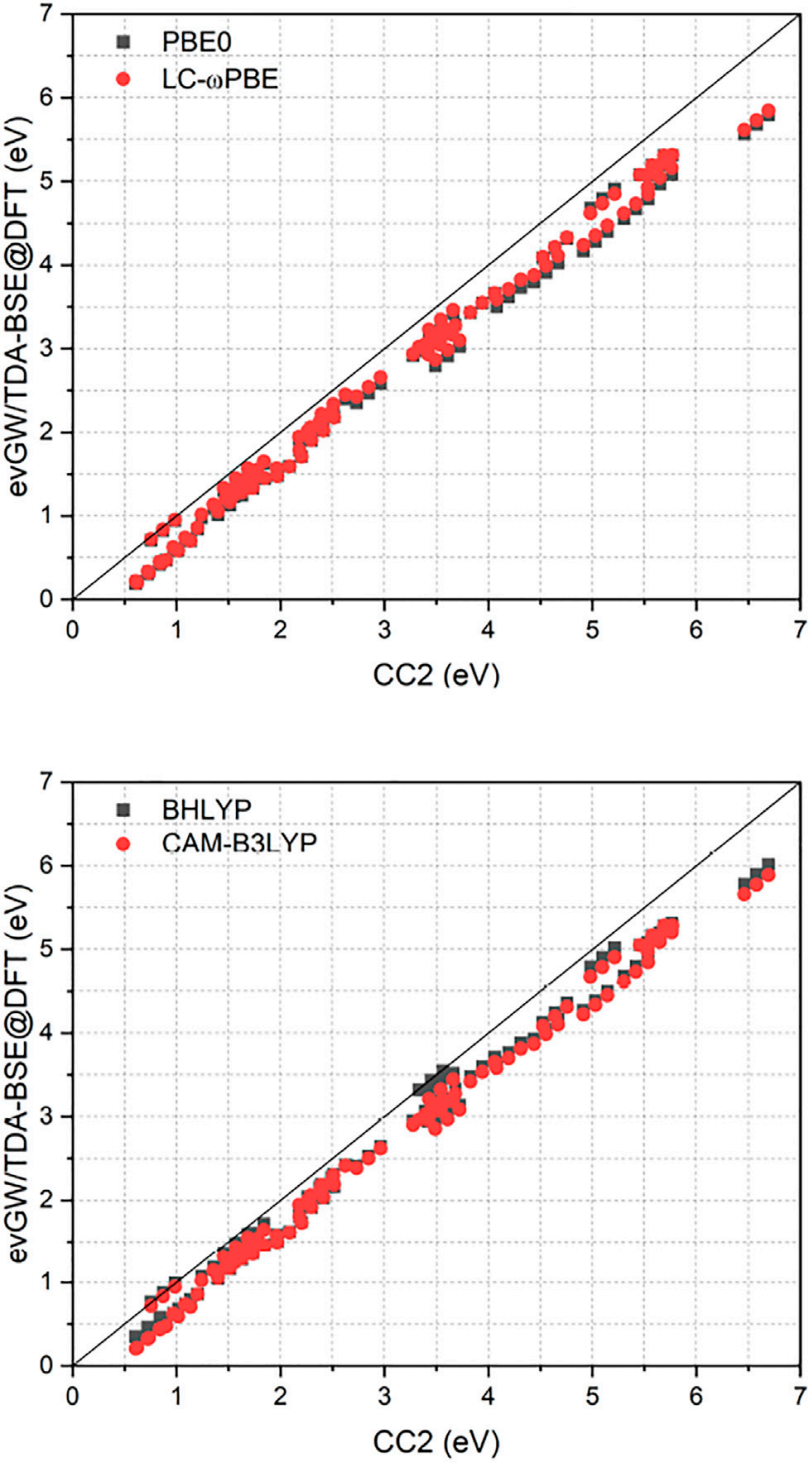
Comparison of excitation energies from the RI-CC2 and ev*GW*/TDA-BSE@DFT methods. ev*GW*/BSE calculations have been performed using either PBE0 or LC-*ω*PBE spinors. All calculations used the def2-TZVP basis set. All values in eV.

Figure 1 compares the RI-CC2 and ev*GW*/BSE excitation energies of the full set. It exhibits a near-linear shift between the two methods, with the difference getting more pronounced for excited states with higher energy. Furthermore, RI-CC2 consistently yields blue-shifted excitation energies when compared to ev*GW*/BSE. This is in accordance with the finding of Suellen *et al.*, who also found CC2 to yield too high excitation energies on average for this test set, though in the field-free case. In contrast, ev*GW*/BSE was found to yield too low excitation energies on average, especially for triplet excited states. (Gui et al., 2018). Too low triplet excited states are well known phenomenon for the *GW*/BSE method. (Rangel et al., 2017; Jacquemin et al., 2017; Holzer and Klopper, 2018). While Jacquemin *et al.* proposed to use the Tamm-Dancoff approximation (TDA) to improve this, (Jacquemin et al., 2017), two of us proposed adding the correlation kernel of the underlying density functional approximation to improve triplet excitations, yielding the correlation-kernel augmented BSE (cBSE) approach. (Holzer and Klopper, 2018). While a linear-response time-dependent DFT implementation in magnetic fields would be needed to apply the cBSE method, using the TDA is straightforwardly obtained by setting **B** = **0** in Eq. 16. For the tested molecules, these findings can be partly confirmed, with the TDA leading to a significantly blue shift of especially the lower lying excited states, improving the agreement between ev*GW*/TDA-BSE and RI-CC2 (Figure 2). The improvement of the *GW*/BSE method when using the TDA is generally also observed in field-free cases. (Rangel et al., 2017).

A closer inspection of Figure 1 reveals that for the ev*GW*/BSE method, two distinct groups, one with smaller deviations from RI-CC2 and one with larger deviations, are found. The “high-error” group is composed of the molecules nitrosamine (1.02 eV), HCP (3.61 eV), diacetylene (4.20 eV), cyanoacetylene (4.55 eV), cyanogen (5.03 eV), isocyanogen (5.42 eV), acetylene (5.65 eV), and HCN (6.58 eV). The values in parenthesis are the T_0_ excitation energies of the corresponding RI-CC2 references. Except for the low-energy excited state of nitrosamine, all these molecules feature triple bonds in their respective ground states. Furthermore we find instabilities for the molecules HNO (0.74 eV) and nitrosylcyanide (0.72 eV) with rather low lying triplet excited states. This is further hinting at triple bonds and nitrosyl groups being described with sub-par quality within the ev*GW*/BSE methods. For the remaining molecules significantly smaller errors are found.

Employing the TDA removes the instabilities encountered in the ev*GW*/BSE calculations for the molecules HNO and nitrosylcyanide, in both cases yielding excitation energies that are lower by ≈0.4 eV when compared to their RI-CC2 counterparts as shown in Table 1.

**TABLE 1:** Mean average error (MAE), mean signed error (MSE), standard deviation (SD), and maximum error (MAX) of ev*GW*/BSE@DFT (“BSE”) and ev*GW*/TDA-BSE@DFT (“TDA”) excitation energies with respect to CC2 excitation energies. All values in eV.

DFT	PBE0		LC-*ω*PBE		BHLYP		CAM-B3LYP	
Method	BSE	TDA	BSE	TDA	BSE	TDA	BSE	TDA
MAE	0.61	0.41	0.58	0.38	0.53	0.34	0.59	0.39
MSE	−0.61	−0.41	−0.58	−0.38	−0.53	−0.34	−0.59	−0.39
SD	0.28	0.18	0.25	0.17	0.23	0.16	0.25	0.16
MAX	1.29	0.90	1.24	0.85	1.05	0.68	1.19	0.81

As displayed also in Figure 1 and Figure 2, Table 1 reveals that BHLYP, which incorporates a relatively large amount of Hartree–Fock exchange, performs best for the investigated molecules. The range-separated hybrids LC-*ω*PBE and CAM-B3LYP yield comparable results, and generally perform better than PBE0 but worse than BHLYP. This is in line with observations for field-free cases, indicating that (at least for moderate field strength) conclusions drawn from field-free benchmarks are still applicable. (Holzer et al., 2021).

As shown in Figure 2, the class of molecules with triple bonds or nitrosyl groups exhibits a significantly reduced error within the TDA for all investigated functionals. Triplet excitation energies from the latter class of molecules are now in line with all other molecules. We therefore expect the TDA to be especially valuable for molecules with triple bonds or nitrosyl groups. Still, regarding the TDA, there are some caveats left. While some of the improvements can indeed be related to error compensation, where the blue-shift of the TDA counteracts the general red-shift of the ev*GW*/BSE method with respect to CC2 excitation energies, this can not fully explain the strong reduction of the error regarding the class of molecules with triple bonds or nitrosyl groups, which indicates that also the correlation from the BSE is sometimes insufficient to describe triplet excitations sufficiently well. Given the overall increase in accuracy from the TDA, it may be advisable to even use it by default in magnetic fields until the cBSE method becomes available. (Holzer and Klopper, 2018). However, it shall be noted that the usage of CC2 as reference method is not the best possible but a pragmatic choice for this test set. While its accuracy is comparable or even slightly better than that of EOM-CCSD, (Suellen et al., 2019), more refined methods as CC3 or EOM-CCSDT would be needed to obtain true reference values with errors significantly below 0.1 eV. Given the immense computational cost of the latter two methods, only results for a single diatomic molecule, namely CH^+^, have been reported for EOM-CCSDT so far in a finite magnetic field. (Hampe et al., 2020). RI-CC2 as computational efficient method is therefore a suitable compromise, providing robust values. However, as shown in Ref. (Suellen et al., 2019), CC2 has a tendency to deliver too high excitation energies when compared to experimental and CC3 excitation energies. In contrast, ev*GW*/BSE tends to underestimate excitation energies as shown in Ref. (Gui et al., 2018), especially for triplet excited states. This has to be taken into account when comparing the CC2 and ev*GW*/BSE methods. Concerning the reference state, ev*GW* is able to even out the differences between the underlying functionals completely. The difference between excitation energies obtained from either ev*GW*/BSE@PBE0 or ev*GW*/BSE@LC-*ω*PBE is statistically insignificant. The presented results suggest that the performance of the ev*GW*/BSE method in magnetic fields is similar to its performance in field-free situations, yielding good to excellent excitation energies, at a considerably reduced effort when compared to coupled-cluster methods.

### 4.2 Optical Properties of Tetracene in a Magnetic Field

As established in the last section, the ev*GW*/BSE method quite accurately predicts molecular excitation energies in the presence of an external magnetic field. Our implementation into the Turbomole package thus appears to be an efficient yet reliable method of predicting excited state properties of sizable molecules in strong magnetic fields. As a consequence, real-world properties such as the absorption and emission spectra, and therefore also the colour, of a substance can now be obtained by simulating the vertical excitations and related oscillator strengths of a molecule under such extreme conditions. In this section, the effects of a strong external magnetic field on the excited states of tetracene are studied in detail, exemplifying the effects such extreme environments can have on chemical substances.

The optical spectrum of tetracene in the absence of an external magnetic field is mainly composed of three bands. The *p*-band (peak at 455–477 nm) corresponds to the HOMO → LUMO transition (B_2u_ symmetry). The *α*- and *β*-bands correspond to the HOMO → LUMO+1 and HOMO −1 → LUMO transitions (both B_3u_ symmetry), respectively, and show peaks at 373–393 nm (*α*-band) as well as 272–275 nm (*β*-band). While the two bands in the visible (*p*) and near-UV (*α*) region of the spectrum exhibit relatively small oscillator strengths, the *β* excitation is associated with an oscillator strength several orders of magnitude larger than that of the two other transitions. (Guidez and Aikens, 2013; Sony and Shukla, 2007; Klevens and Platt, 1949; Biermann and Schmidt, 1980; Bree and Lyons, 1960).

In order to investigate the optical properties of tetracene for the field-free case, we first optimized the geometry at the PBE0, B3LYP, BHLYP and CAM-B3LYP levels, respectively. Using these structures, subsequent CD-ev*GW*/BSE@DFT calculations were carried out. The resulting wavelengths for the excitations are presented in Table 2. The different reference functionals provide similar values for the *α*- and *β*-excitations, slightly overestimating the energies of both excitations. The energies of the *p*-excitation, however, vastly differ with respect to the reference functional, ranging from 465 nm at the CD-ev*GW*/BSE@BHLYP level to 614 nm at the CD-ev*GW*/BSE@PBE0 level. Further investigations reveal that this is almost exclusively an effect of the geometry and not the method itself as using the CAM-B3LYP reference geometry yields wavelengths between 468 and 487 nm for the *p*-excitation in all cases. This is to be expected as the impact of the reference functional is usually not very large for ev*GW*/BSE calculations. (Holzer et al., 2021).

**TABLE 2:** Wavelengths of *p*-, *α*- and *β*-excitations in nm as calculated at the CD-ev*GW*/BSE@DFT level employing different reference functionals. In order to highlight the dependence on the geometry, all calculations were performed both on the geometry as optimized using the reference functional as well as on the geometry as optimized using the range-separated hybrid (RSH) functional CAM-B3LYP. Experimental values are also given.

Geometry	Geometry optimization			CAM-B3LYP (RSH) geometry				
Functional	PBE0	B3LYP	BHLYP	RSH	PBE0	B3LYP	BHLYP	Exp. Guidez and Aikens, (2013); Sony and Shukla, (2007); Klevens and Platt, (1949); Biermann and Schmidt, (1980); Bree and Lyons, (1960)
*p*-Excitation	614	505	465	470	484	487	468	455–477
*α*-Excitation	370	369	349	354	359	360	351	373–393
*β*-Excitation	270	269	260	265	264	264	261	272–275

**FIGURE 3:**
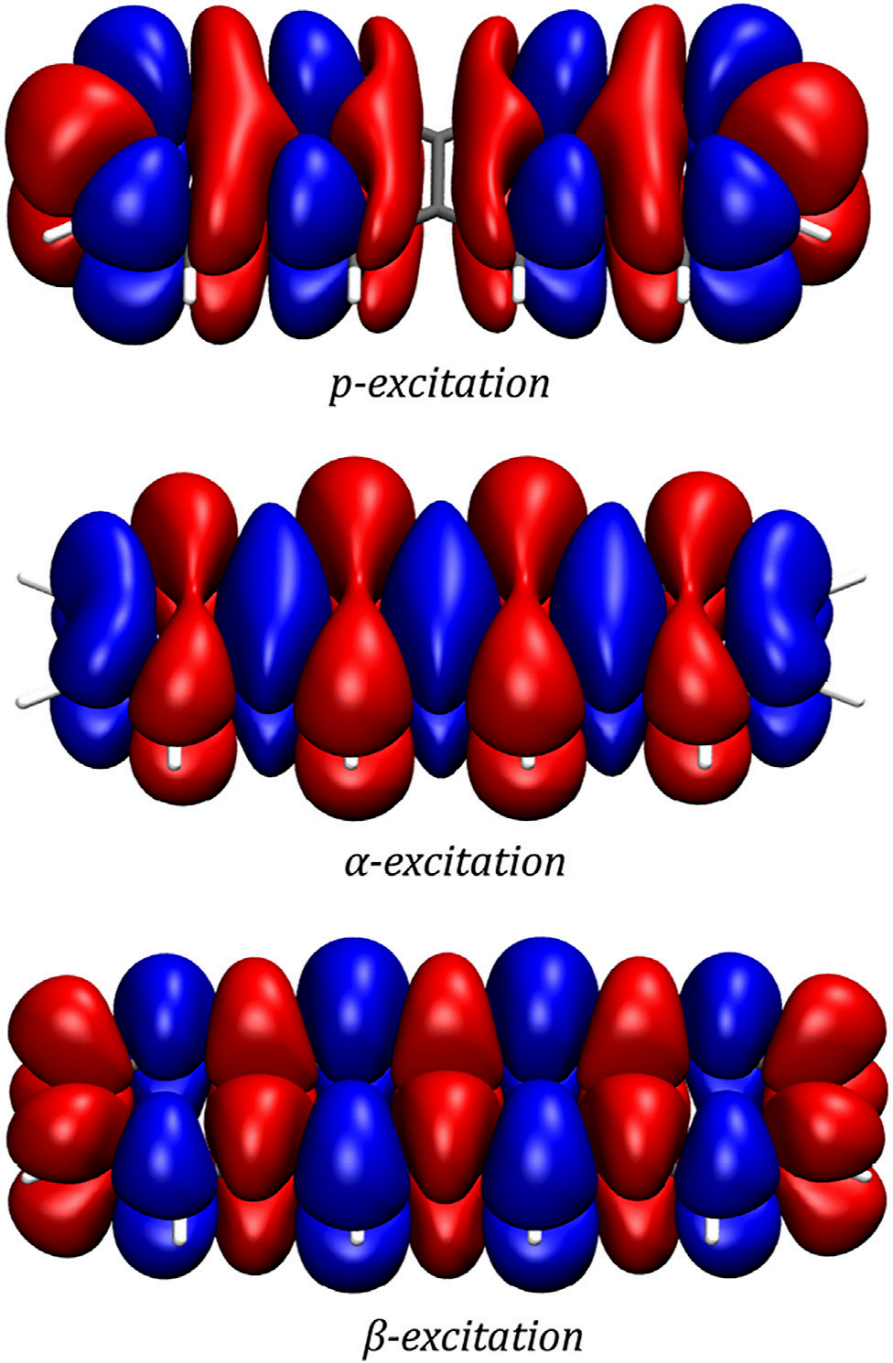
Transition densities of the *p*-, *α*- and *β*-excitations as generated by a two-component CD-ev*GW*/BSE@CAM-B3LYP calculation. The plots were generated with VMD using an isovalue of 0.0001 $a_{0}^{-3}$ for the *p*-excitation and 0.0002 $a_{0}^{-3}$ for the *α*- and *β*-excitations. The colour blue indicates a loss while red indicates a gain of electron density.

In order to gain a better insight into the electronic processes behind the optical spectrum, the transition densities are examined for the *p*-, *α*- and *β*-excitations. In accordance with the literature, (Lim et al., 2004; Guidez and Aikens, 2013), it is found that while the *p*-band corresponds to a transition polarized along the short axis of the molecule, the *α*- and *β*-excitations can be described by transitions polarized along the long axis of the molecule. The transition densities of the three excitations are depicted in Figure 3.

Having gained a general overview of the electronic excitations that mainly constitute the optical spectrum of tetracene, it is now possible to extend these findings in order to understand the effect a strong external magnetic field may have on such a system. Furthermore, it is now possible to use these findings in order to predict the colour shift of the tetracene molecule under the influence of such a strong magnetic field.

This investigation contains three steps: Firstly, it is necessary to generate the optical spectrum of tetracene in the absence of a magnetic field. This was done using the CD-ev*GW* (10)/BSE@DFT methods employing BHLYP and CAM-B3LYP as reference functionals as they most accurately describe the electronic excitations in the zero-field compared to the experimental values. The peaks are broadened using a damped sum-over-states formalism which translates to a Lorentzian line shape with full width at half maximum of 0.15 eV for all excitations. (Norman et al., 2004; Barron, 2004; Fernandez-Corbaton et al., 2020). From this information, the RGB colour code of the substance can be computed. By calculating the integrals over the entire visible part of the spectrum and arbitrarily setting it to 1 for the zero-field case, the relative intensities of the colour may also be calculated.

Secondly, the immediate influence of an external magnetic field has to be assessed. Applying the external field perpendicular to the molecular plane lowers the symmetry of the system. The point group of tetracene in such an external field is C_2h_ instead of D_2h_. The excitations of the *p*-, *α*- and *β*-bands are all of B_u_ symmetry. A further investigation reveals that the subsequent two excitations (here denoted as *γ* and *δ*) are also of B_u_ symmetry.

**FIGURE 4:**
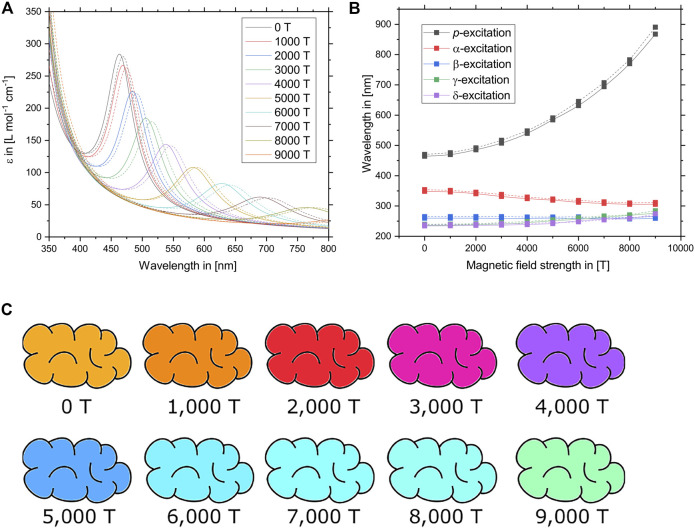
**(A)** UV/Vis spectra of tetracene as predicted at different magnetic field strengths between 0 T and 9,000 T. Solid lines denote calculations using the CD-ev*GW* (10)/BSE@BHLYP method while dashed lines denote CD-ev*GW* (10)/BSE@CAM-B3LYP calculations. **(B)** Wavelengths of the relevant lowest vertical excitations of tetracene at different magnetic field strengths between 0 T and 9,000 T. The *p*-excitation, which is predominantly responsible for the colour of tetracene, is most affected by the external field. Solid lines denote calculations using the CD-ev*GW* (10)/BSE@BHLYP method while dashed lines denote CD-ev*GW* (10)/BSE@CAM-B3LYP calculations. **(C)** Colour of tetracene as predicted at the CD-ev*GW* (10)/BSE@CAM-B3LYP level of theory at different magnetic field strengths between 0 T and 9,000 T. To obtain the depicted colours, the vertical excitations of the optical spectrum were broadened by 0.15 eV and converted into a RGB colour code while the intensity was scaled relative to the zero-field by integrating over the visible region of the spectrum.

Thirdly, by slowly raising the magnetic field strength in steps of 1,000 T and generating the optical spectrum at each field strength as previously described, it is possible to track how the excitations are influenced by the external magnetic field. The resulting UV/Vis spectra and the energies of the five lowest excitations are plotted in Figures 4A,B. While the excitation energies of the *α*–*δ*-excitations are only slightly shifted between 0 T and 9,000 T, the *p*-band is strongly red-shifted. At the same time, the oscillator strength of the *p*-excitation decreases with an increasing field strength. The resulting UV/Vis spectrum is mostly dominated by both the location and intensity of the *p*-band while only a small section of the violet and blue part of the spectrum between 400 and 500 nm is caused by the other excitations. Only the five lowest excitations (*p*, *α*–*δ*) are depicted in Figure 4B, but the UV/Vis spectrum was generated by the 75 lowest excitations. However, none of the other excitations contribute significantly to the visible part of the spectrum.

The resulting predicted colours of tetracene at various magnetic field strengths are shown in Figure 4C. As the *p*-band is red-shifted, the main absorption band moves from the blue part of the spectrum towards the red. The orange-red colour of tetracene therefore shifts towards the colour blue. Finally, between 5,000 T and 9,000 T, the contribution of the *p*-band does not contribute significantly to the spectrum anymore. Since the *α*–*δ*-bands are still active and relatively unchanged in their location, part of the blue light is still absorbed, resulting in a turquoise to green colour which becomes less and less intense as the magnetic field strength is increased.

It is worth noting that while the peak positions of the *α*–*δ*-excitations exhibit only a minor dependence on the applied field, their respective oscillator strengths change significantly as the *β*-excitation becomes less important. Subsequently, the *γ*- and *δ*-excitations become dominant at different magnetic field strengths. Furthermore, certain additional transitions slowly start to arise as they are no longer symmetry forbidden due to a lowering of the point group symmetry in the magnetic field. At a magnetic field strength of approximately 6,000 T, specifically, the *γ*- and *δ*-excitations are very close energetically to the usually predominant *β*-excitation, leading to resonance phenomena such as a splitting into multiplets.

Finally, the strong influence of the external field on the excitation energy of the *p*-band can best be understood by examining the changes to the electronic structure of tetracene. In the magnetic field, the energy of the HOMO increases and the energy of the LUMO decreases. Thus, the HOMO-LUMO gap decreases significantly. As the *p*-excitation corresponds to the HOMO → LUMO transition, the resulting excitation energy is subsequently lowered.

**FIGURE 5:**
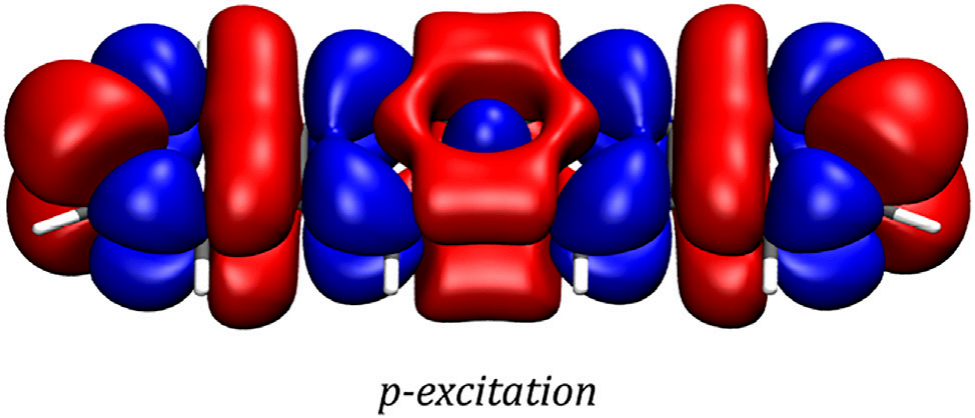
Transition density of the *p*-excitation at a magnetic field strength of 8,000 T as generated by a two-component CD-ev*GW*/BSE@CAM-B3LYP calculation. The plot was generated with VMD using an isovalue of 0.0001 $a_{0}^{-3}$ . The colour blue indicates a loss while red indicates a gain of electron density.

The transition density of the *p*-excitation at a magnetic field strength of 8,000 T is depicted in Figure 5. It exhibits the effects the magnetic field has on this most important transition, showing a slightly more delocalized nature of this transition in the magnetic field compared to the zero-field case. In order to ensure gauge-origin invariance, the transition density plot was generated employing London atomic orbitals.

## 5. Conclusion

In this paper, we have presented a gauge-invariant formulation of the *GW*/BSE method for excited states in strong magnetic fields. The resulting implementation was benchmarked against reference values obtained from approximate coupled-cluster (CC2) theory. The obtained results indicate that the *GW*/BSE method provides a similar accuracy in strong magnetic fields as in the field-free case. The known issue of an underestimation of the excitation energy of triplet excited states is also present in magnetic fields. Like in the field-free case, it is shown that the Tamm-Dancoff approximation is able to remove a significant amount of this underestimation, improving the overall accuracy when compared to coupled-cluster values. The remaining error is nearly linear, making it easy to be accounted for.

Furthermore, using the tetracene molecule as showcase example, it was demonstrated that the *GW*/BSE method is able to tackle systems far beyond the possibilities of any prior ansatz that has been used to describe excited states in strong magnetic fields. For the tetracene molecule, we analyzed the shift of the main absorption peaks in magnetic fields ranging from 0 to 9,000 T. It was found that some excited state energies are more affected than others, leading to prominent changes in the spectrum. Ultimately, the colour of tetracene was estimated from the calculated spectra in the assessed magnetic fields. Starting from the bright orange colour of tetracene, we predict the compound to exhibit a blue colour at 5,000, which is converted towards a green colour at 9,000 T. While the dependence of the excited states on the external magnetic field are interesting on their own, the example of tetracene also outlines the fascinating world that even moderately strong magnetic fields could open for the broad field of photochemistry.

To summarize, the *GW*/BSE method has proven once more that it has become a formidable member of the toolbox of quantum chemistry.

## Data Availability

The original contributions presented in the study are included in the article/Supplementary Material, further inquiries can be directed to the corresponding author.
