# Supplementary material for: The GW/BSE Method in Magnetic Fields
Source: Front Chem. 2021 Nov 25;9:746162. doi: 10.3389/fchem.2021.746162 (PMC8655096; doi:10.3389/fchem.2021.746162)
Supplement: Supplementary file 1 [file DataSheet1.zip › Data Sheet 1/HolzerEtAlSupportingInformation1.pdf]

Supporting Information: The GW/BSE method in magnetic fields

Christof Holzer,† Ansgar Pausch,‡ and Wim Klopper*,‡,¶

†Institute of Theoretical Solid State Physics, Karlsruhe Institute of Technology (KIT), Wolfgang-Gaede-Straße 1, 76131 Karlsruhe, Germany

‡Institute of Physical Chemistry, Karlsruhe Institute of Technology (KIT), Fritz-Haber-Weg 2, 76131 Karlsruhe, Germany

¶Institute of Nanotechnology, Karlsruhe Institute of Technology (KIT), Hermann-von-Helmholtz-Platz 1, 76344 Eggenstein-Leopoldshafen, Germany

E-mail: klopper@kit.edu

S1 Excitation energies for a set of small molecules with RI-CC2 and GW/BSE in a magnetic field

This section lists the obtained RI-CC2, evGW/BSE and evGW/TDA-BSE excitation energies for the 36 molecules described in the main manuscript. In the evGW calculations all quasiparticle (QP) energies were fully optimized, and the calculation was considered to be converged when the difference in QP energies in two subsequent iterations was less than 10^{-5} Hartree. From the obtained QP energies, then subsequently the excitation energies were obtained using the Bethe-Salpeter equation (BSE), or the Tamm-Dancoff approximation of the BSE (TDA-BSE). This procedure has been repeated for the PBE0, LC-ωPBE, BHLYP, and CAM-B3LYP density funcitonal approximations, representing popular density functional approximations in GW theory. Additionally, as reference, the RI-CC2 excitation energies have been calculated for the given molecules.

All calculations were performed in a static external magnetic field of 1000 Tesla. The def2-TZVP basis set was used throughout. The obtained results are listed in Tab. S1 and S2. For the molecules HNO and nitrosylcyanide the evGW/BSE calculations exhibited an artificial instability, that can be removed using the TDA.

Table S1: RI-CC2 and evGW/BSE excitation energies for the first split triplet of various molecules. evGW/BSE calculation used fully optimized evGW quasiparticle energies. evGW/BSE calculations have been performed for the PBE0, LC-ωPBE (ωPBE), BHLYP, and CAM-B3LYP (CAM) functionals. All calculations used the def2-TZVP basis set. All values in eV

	RI-CC2	evGW/BSE@DFT			
Molecule		PBE0	ωPBE	BHLYP	CAM
Acetaldehyd	3.83	3.37	3.37	3.42	3.35
	3.94	3.48	3.49	3.54	3.47
	4.06	3.60	3.60	3.65	3.59
Acetylene	5.53	4.48	4.57	4.75	4.62
	5.65	4.60	4.69	4.87	4.73
	5.76	4.71	4.80	4.98	4.85
CCl_2	0.75	0.21	0.25	0.33	0.23
	0.87	0.33	0.37	0.45	0.35
	0.99	0.44	0.48	0.56	0.46
CClF	1.45	0.93	0.99	1.01	0.96
	1.57	1.05	1.11	1.13	1.08
	1.69	1.16	1.22	1.25	1.19
CF_2	2.39	1.83	1.90	1.85	1.85
	2.51	1.95	2.02	1.97	1.97
	2.63	2.06	2.13	2.09	2.09
Cyanoacetylene	4.44	3.37	3.48	3.55	3.46
	4.55	3.49	3.59	3.67	3.58
	4.67	3.61	3.71	3.78	3.70
Cyanoformaldehyde	3.27	2.84	2.86	2.88	2.83
	3.39	2.96	2.98	3.00	2.95
	3.51	3.07	3.09	3.11	3.06
Cyanogen	4.91	3.73	3.81	3.87	3.79
	5.03	3.85	3.93	3.98	3.91
	5.15	3.96	4.04	4.10	4.02
Diacetylene	4.08	3.09	3.20	3.29	3.19
	4.20	3.20	3.32	3.40	3.31
	4.31	3.32	3.44	3.52	3.42
Difluorodiazirine	2.73	2.26	2.34	2.32	2.29

Table S1: continued. All values in eV

Molecule	RI-CC2	evGW/BSE@DFT PBE0	ωPBE	BHLYP	CAM
	2.85	2.37	2.45	2.44	2.41
	2.96	2.49	2.57	2.55	2.53
Formaldehyde	3.45	2.98	2.98	3.04	2.97
	3.56	3.10	3.10	3.15	3.09
	3.68	3.22	3.21	3.27	3.20
Formic acid	5.46	5.02	5.01	4.99	4.99
	5.57	5.13	5.13	5.11	5.10
	5.69	5.25	5.25	5.22	5.22
Formyl chloride	4.52	4.01	4.03	4.06	4.01
	4.64	4.13	4.14	4.17	4.12
	4.76	4.24	4.26	4.29	4.24
Formyl fluoride	5.54	5.01	5.01	4.98	4.98
	5.65	5.12	5.13	5.10	5.10
	5.77	5.24	5.25	5.21	5.21
Glyoxal	2.26	1.91	1.94	1.97	1.92
	2.38	2.02	2.05	2.08	2.04
	2.49	2.14	2.17	2.20	2.15
H_2C_3	1.24	0.89	0.93	0.99	0.92
	1.36	1.00	0.95	1.04	0.94
	1.47	1.12	1.05	1.11	1.04
HCN	6.46	5.17	5.23	5.42	5.27
	6.58	5.29	5.34	5.54	5.39
	6.69	5.40	5.46	5.65	5.50
HCP	3.49	2.40	2.45	2.55	2.48
	3.61	2.52	2.57	2.66	2.59
	3.72	2.63	2.68	2.78	2.71
HPO	1.55	1.04	1.06	1.05	1.05
	1.67	1.16	1.18	1.17	1.17
	1.78	1.28	1.30	1.29	1.28
HPS	0.97	0.43	0.45	0.45	0.45
	1.08	0.55	0.56	0.57	0.56

Table S1: continued. All values in eV

Molecule	RI-CC2	evGW/BSE@DFT PBE0	ωPBE	BHLYP	CAM
	1.20	0.66	0.68	0.68	0.68
HSiF	1.54	0.95	1.02	0.95	1.00
	1.66	1.07	1.14	1.06	1.12
	1.78	1.18	1.25	1.18	1.23
Isocyanogen	5.30	4.18	4.26	4.34	4.25
	5.42	4.30	4.37	4.46	4.37
	5.54	4.41	4.49	4.57	4.48
Nitrosamine	0.90	0.02	0.02	0.25	0.04
	1.02	0.14	0.14	0.36	0.16
	1.13	0.25	0.25	0.48	0.28
Phosgene	4.98	4.61	4.55	4.72	4.60
	5.10	4.72	4.66	4.84	4.72
	5.21	4.84	4.78	4.95	4.83
Propynal	3.33	2.90	2.96	3.25	2.90
	3.45	3.02	3.07	3.37	3.01
	3.56	3.13	3.19	3.40	3.13
Pyrazine	3.43	3.13	3.16	3.22	3.14
	3.54	3.24	3.28	3.34	3.26
	3.66	3.36	3.40	3.45	3.38
Selenoformaldehyde	1.40	0.93	0.97	0.97	0.97
	1.51	1.05	1.09	1.08	1.09
	1.63	1.16	1.20	1.20	1.20
$SiCl_2$	2.18	1.70	1.72	1.67	1.72
	2.29	1.81	1.83	1.79	1.83
	2.41	1.93	1.95	1.91	1.95
Silylidene	1.97	1.42	1.43	1.45	1.44
	2.09	1.54	1.54	1.57	1.56
	2.20	1.65	1.66	1.68	1.67
Tetrazine	1.61	1.29	1.32	1.39	1.30
	1.72	1.41	1.43	1.51	1.42
	1.84	1.52	1.55	1.62	1.54

Table S1: continued. All values in eV

Molecule	RI-CC2	evGW/BSE@DFT			
		PBE0	ωPBE	BHLYP	CAM
Thioformaldehyde	1.73	1.24	1.25	1.27	1.26
	1.85	1.36	1.36	1.39	1.37
	1.96	1.48	1.48	1.50	1.49
Thioformylchloride	2.18	1.71	1.72	1.72	1.73
	2.30	1.82	1.83	1.84	1.84
	2.41	1.94	1.95	1.95	1.96
Thionylcarbonylfluoride	3.42	2.89	2.87	2.87	2.88
	3.54	3.01	2.98	2.98	3.00
	3.65	3.12	3.10	3.05	3.07
Thiophosgene	2.29	1.88	1.87	1.86	1.88
	2.40	1.99	1.99	1.98	2.00
	2.52	2.11	2.11	2.09	2.11

Table S2: RI-CC2 and evGW/TDA-BSE excitation energies for the first split triplet of various molecules. evGW/TDA-BSE calculation used fully optimized evGW quasiparticle energies. evGW/TDA-BSE calculations have been performed for the PBE0, LC-ωPBE (ωPBE), BHLYP, and CAM-B3LYP (CAM) functionals. All calculations used the def2-TZVP basis set. All values in eV

	RI-CC2	evGW/TDA-BSE@DFT			
Molecule		PBE0	ωPBE	BHLYP	CAM
Acetaldehyd	3.83	3.43	3.37	3.48	3.42
	3.94	3.55	3.49	3.60	3.53
	4.06	3.66	3.60	3.71	3.65
Acetylene	5.53	4.85	4.57	5.08	4.97
	5.65	4.97	4.69	5.20	5.09
	5.76	5.08	4.80	5.31	5.20
CCl_2	0.75	0.71	0.25	0.76	0.72
	0.87	0.83	0.37	0.88	0.83
	0.99	0.94	0.48	1.00	0.95
CClF	1.45	1.30	0.99	1.36	1.32
	1.57	1.42	1.11	1.48	1.43
	1.69	1.53	1.22	1.59	1.55
CF_2	2.39	2.16	1.90	2.19	2.18
	2.51	2.28	2.02	2.31	2.30
	2.63	2.40	2.13	2.42	2.41
Cyanoacetylene	4.44	3.80	3.48	3.93	3.87
	4.55	3.91	3.59	4.05	3.98
	4.67	4.03	3.71	4.16	4.10
Cyanoformaldehyde	3.27	2.92	2.86	2.95	2.90
	3.39	3.03	2.98	3.07	3.02
	3.51	3.15	3.09	3.18	3.13
Cyanogen	4.91	4.17	3.81	4.27	4.22
	5.03	4.28	3.93	4.39	4.33
	5.15	4.40	4.04	4.50	4.45
Diacetylene	4.08	3.50	3.20	3.65	3.58
	4.20	3.62	3.32	3.77	3.70
	4.31	3.73	3.44	3.88	3.81
Difluorodiazirine	2.73	2.35	2.34	2.41	2.39

Table S2: continued. All values in eV

Molecule	RI-CC2	evGW/TDA-BSE@DFT PBE0	ωPBE	BHLYP	CAM
	2.85	2.47	2.45	2.53	2.50
	2.96	2.58	2.57	2.64	2.62
Formaldehyde	3.45	3.05	2.98	3.10	3.04
	3.56	3.17	3.10	3.22	3.16
	3.68	3.29	3.21	3.33	3.27
Formic acid	5.46	5.08	5.01	5.05	5.05
	5.57	5.20	5.13	5.17	5.17
	5.69	5.31	5.25	5.29	5.28
Formyl chloride	4.52	4.09	4.03	4.13	4.08
	4.64	4.20	4.14	4.24	4.20
	4.76	4.32	4.26	4.36	4.31
Formyl fluoride	5.54	5.08	5.01	5.05	5.05
	5.65	5.19	5.13	5.17	5.17
	5.77	5.31	5.25	5.28	5.28
Glyoxal	2.26	1.99	1.94	2.04	2.00
	2.38	2.10	2.05	2.16	2.11
	2.49	2.22	2.17	2.27	2.23
H_2C_3	1.24	0.98	0.93	1.07	1.02
	1.36	1.09	0.95	1.19	1.14
	1.47	1.15	1.05	1.28	1.18
HCN	6.46	5.57	5.23	5.78	5.66
	6.58	5.68	5.34	5.90	5.77
	6.69	5.80	5.46	6.01	5.89
HCP	3.49	2.79	2.45	2.91	2.85
	3.61	2.91	2.57	3.02	2.97
	3.72	3.03	2.68	3.14	3.08
HNO	0.62	0.22	0.12	0.30	0.21
	0.74	0.34	0.12	0.42	0.33
	0.85	0.45	1.35	0.53	0.45
HPO	1.55	1.23	1.06	1.25	1.23
	1.67	1.35	1.18	1.37	1.35

Table S2: continued. All values in eV

Molecule	RI-CC2	evGW/TDA-BSE@DFT PBE0	ωPBE	BHLYP	CAM
	1.78	1.46	1.30	1.48	1.47
HPS	0.97	0.61	0.45	0.63	0.62
	1.08	0.73	0.56	0.74	0.74
	1.20	0.84	0.68	0.86	0.85
HSiF	1.54	1.27	1.02	1.27	1.30
	1.66	1.38	1.14	1.39	1.42
	1.78	1.50	1.25	1.50	1.53
Isocyanogen	5.30	4.56	4.26	4.68	4.61
	5.42	4.67	4.37	4.80	4.73
	5.54	4.79	4.49	4.91	4.84
Nitrosamine	0.90	0.47	0.02	0.57	0.47
	1.02	0.59	0.14	0.68	0.59
	1.13	0.70	0.25	0.80	0.71
Nitrosylcyanide	0.61	0.19	0.12	0.35	0.20
	0.72	0.30	0.12	0.47	0.32
	0.84	0.42	1.30	0.58	0.44
Phosgene	4.98	4.68	4.55	4.79	4.67
	5.10	4.80	4.66	4.91	4.79
	5.21	4.91	4.78	5.02	4.90
Propynal	3.33	2.97	2.96	3.32	2.96
	3.45	3.09	3.07	3.44	3.08
	3.56	3.20	3.19	3.55	3.20
Pyrazine	3.43	3.19	3.16	3.29	3.21
	3.54	3.31	3.28	3.40	3.33
	3.66	3.42	3.40	3.52	3.44
Selenoformaldehyde	1.40	1.01	0.97	1.04	1.05
	1.51	1.13	1.09	1.16	1.17
	1.63	1.24	1.20	1.28	1.28
$SiCl_2$	2.18	1.93	1.72	1.91	1.94
	2.29	2.05	1.83	2.03	2.06
	2.41	2.16	1.95	2.14	2.18

Table S2: continued. All values in eV

Molecule	RI-CC2	evGW/TDA-BSE@DFT PBE0	ωPBE	BHLYP	CAM
Silylidene	1.97	1.48	1.43	1.51	1.49
	2.09	1.59	1.54	1.62	1.61
	2.20	1.71	1.66	1.74	1.73
Tetrazine	1.61	1.40	1.32	1.49	1.41
	1.72	1.51	1.43	1.60	1.52
	1.84	1.63	1.55	1.72	1.64
Thioformaldehyde	1.73	1.33	1.25	1.35	1.34
	1.85	1.44	1.36	1.47	1.46
	1.96	1.56	1.48	1.58	1.57
Thioformylchloride	2.18	1.79	1.72	1.80	1.80
	2.30	1.90	1.83	1.91	1.92
	2.41	2.02	1.95	2.03	2.03
Thionylcarbonylfluoride	3.42	2.97	2.87	2.94	2.96
	3.54	3.08	2.98	3.06	3.08
	3.65	3.20	3.10	3.18	3.19
Thiophosgene	2.29	1.95	1.87	1.93	1.95
	2.40	2.07	1.99	2.05	2.07
	2.52	2.18	2.11	2.16	2.19
